# Extremely Low Frequency-Magnetic Field (ELF-MF) Exposure Characteristics among Semiconductor Workers

**DOI:** 10.3390/ijerph15040642

**Published:** 2018-03-31

**Authors:** Sangjun Choi, Wonseok Cha, Jihoon Park, Seungwon Kim, Won Kim, Chungsik Yoon, Ju-Hyun Park, Kwonchul Ha, Donguk Park

**Affiliations:** 1 Department of Occupational Health, Daegu Catholic University, Gyeongsan 38430, Korea; junilane@gmail.com; 2 Department of Environmental Health, Korea National Open University, Seoul 03087, Korea; aromaticwind@gmail.com; 3 Department of Environmental Health Sciences, Institute of Health and Environment, Graduate School of Public Health, Seoul National University, Seoul 08826, Korea; jhabso@nate.com (J.P.); csyoon@snu.ac.kr (C.Y.); 4 Department of Public Health, Keimyung University, Daegu 42601, Korea; swkim@kmu.ac.kr; 5 Wonjin Institute of Occupational and Environmental Health, Seoul 02221, Korea; gganna@hanmail.net; 6 Department of Statistics, Dongguk University, Seoul 04620, Korea; jujuhyunp@gmail.com; 7 Department of Biochemistry and Health Science, Changwon National University, Changwon 51140, Korea; kcha@changwon.ac.kr

**Keywords:** extremely low frequency-magnetic fields (ELF-MF), fabrication (fab) and chip packaging assembly, semiconductors

## Abstract

We assessed the exposure of semiconductor workers to extremely low frequency-magnetic fields (ELF-MF) and identified job characteristics affecting ELF-MF exposure. These were demonstrated by assessing the exposure of 117 workers involved in wafer fabrication (fab) and chip packaging wearing personal dosimeters for a full shift. A portable device was used to monitor ELF-MF in high temporal resolution. All measurements were categorized by operation, job and working activity during working time. ELF-MF exposure of workers were classified based on the quartiles of ELF-MF distribution. The average levels of ELF-MF exposure were 0.56 µT for fab workers, 0.59 µT for chip packaging workers and 0.89 µT for electrical engineers, respectively. Exposure to ELF-MF differed among types of factory, operation, job and activity. Workers engaged in the diffusion and chip testing activities showed the highest ELF-MF exposure. The ELF-MF exposures of process operators were found to be higher than those of maintenance engineers, although peak exposure and/or patterns varied. The groups with the highest quartile ELF-MF exposure level are operators in diffusion, ion implantation, module and testing operations, and maintenance engineers in diffusion, module and testing operations. In conclusion, ELF-MF exposure among workers can be substantially affected by the type of operation and job, and the activity or location.

## 1. Introduction

In South Korea, a total of 18 former semiconductor workers who were involved in several different fabrications (hereafter fab) or chip assembly operations at two large companies have been compensated for occupational diseases as of July 2017 (leukemia = 4, aplastic anemia = 3, brain cancer = 2, breast cancer = 2, lung cancer = 2, malignant lymphoma = 2, polyneuropathy = 1, multiple sclerosis = 1, ovarian cancer = 1). There have been many semiconductor workers with chronic diseases, including cancer and rare diseases, who registered with a government compensation program. There have been no consistent specific operations or agents known to cause the compensated occupational diseases (unpublished results, submitted).

The association between exposure to extremely low frequency-magnetic fields (ELF-MF) and adverse health effects, including several cancers such as brain, leukemia and breast cancer has been widely discussed [1,2,3,4,5]. The International Agency for Research on Cancer (2002) and World Health Organization (2007) classified ELF-MF as possibly carcinogenic to humans (Group 2B), based on studies of childhood leukemia, but inadequate evidence is available for adult cancers [6,7].

Several studies to date have assessed exposure to ELF-MF in fabrication (fab) operations in the semiconductor industry [8]. No epidemiologic studies conducted on the semiconductor industry to date have assessed ELF-MF exposure in order to evaluate chronic health effects, including cancer risk. It is common for semiconductor workers to be exposed to ELF-MF generated from the various electric devices operating on 50/60 Hz electric power used in the semiconductor industry. This study aimed to assess the exposure of semiconductor workers to ELF-MF, identify operational and work factors affecting ELF-MF exposure and suggest an approach for ELF-MF exposure assessment in epidemiologic studies.

## 2. Materials and Methods

### 2.1. Study Plant

We conducted ELF-MF exposure assessment at a single semiconductor company operating 15 wafer fabs and two chip packaging plants. This semiconductor manufacturing company operates two continuous series of discrete operations separately at different plants (sites); the fab operation for the development of dies (also known as dice) and the chip packaging operation to produce chips for final electronic products. Both the oldest 200 mm wafer fab plant and a chip packaging plant were selected for ELF-MF exposure assessment.

### 2.2. Overview of Semiconductor Operations

Semiconductor manufacturing can be categorized into three continuous series of discrete operations that are likely operated separately at distinct sites: the substrate manufacture operation making wafers; the integrated circuit fab operation for the development of dies (or dice), i.e., the individual integrated circuits or discrete devices on a wafer; and the assembly and packaging of these dies to produce the chips for final electronic products [9,10].

The fab of integrated circuits or chips is processed separately in so-called fab clean rooms. Although a fab room is also called a clean room, in fact, the “cleanliness” is intended to prevent contamination of the product, not to promote human health. Fab workers can be exposed to low levels of complex chemicals that are re-circulated from the various operations. Integrated circuits are fabricated onto a silicon wafer through a series of repetitive processes composed of four main operation groups:

- Patterning—oxidation, photolithography, developing, etching, and stripping
- Junction formation—diffusion and ion implantation
- Deposition—epitaxial or chemical vapor deposition
- Metallization—sputtering and evaporation

Wafers are subjected to these steps multiple times in the fab operation as they alternately add and then selectively remove materials in layers from the surface of the wafer to create the different components of the completed integrated circuit. In fab operations, the wafers pass many times in and out of basic operations such as layering, pattering and doping, etc. After the integrated circuitry has been completed in the fab operation, the wafers leave the clean room for the chip packaging operations in which the dies on each wafer are separated into individual dies by sawing or scribe-and-break techniques. Each die is attached to the die-attach area of the package by either a gold-silicon eutectic layer or an epoxy adhesive material. Thin wires are bonded between the chip bonding pads and the inner leads of the package. The separated units referred to as chips are burn-in tested and packaged individually through one of a wide variety of techniques used for electronic products. Chip packaging is a once-through process. The principle of semiconductor operations and the major health hazards generated in these operations has been comprehensively described elsewhere [11,12]. Numerous electric devices generating ELF-MF, including diffusion furnaces, ion implanters, etchers, testers, sputters, and more, are widely used in semiconductor manufacturing industry.

### 2.3. ELF-MF Exposure Assessment Strategy

Portable EMDEX lite and standard EMDEX II meters (Enertech Consultants Inc., Patterson, CA, USA) were used to monitor the level of ELF-MF generated during semiconductor operations. These dosimeters have a flat frequency response in the range of 40–1000 Hz (EMDEX lite) or 800 Hz (EMDEX II) and can monitor from 0.01 µT to 70 µT (EMDEX lite) or 300 µT (EMDEX II) with a resolution of 0.01 µT. The EMDEX-II and EMDEX lite ELF-MF meters used in this study were sent to Enertech and calibrated by them regularly. All our measurements were taken within the certified effective calibration period, which is suggested by the manufacturer to be one year. The EMDEX measures the magnetic field as the magnetic flux density or B-field, which is the exposure metric traditionally used by health studies at power frequency [13].

**Figure 1:**
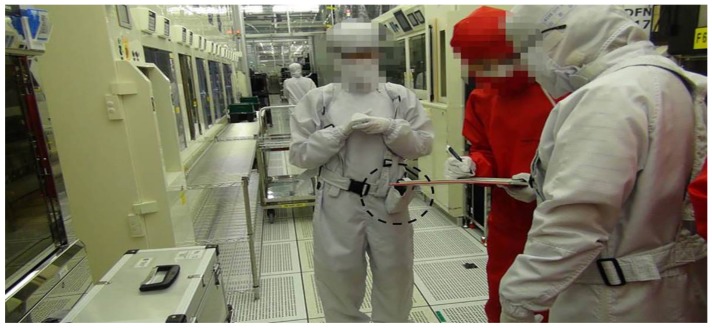
Semiconductor worker wearing portable ELF-MF meter on the waist, indicated with dashed black circle.

A total of 117 semiconductor workers who worked with different devices in different operations of one fab and one chip operation within the semiconductor plant wore personal dosimeters for one full shift (longer than 6 h). These monitors were placed in waist pouches and worn around the hip on the front side of the body (Figure 1). All participants were contacted by investigators shortly after the measurement period and asked to fill out a task activity diary (TAD) we developed and clarify the situation so that the ELF-MF record, logged every four seconds in order to obtain high resolution exposure data could be coded by type of operation, the work activity they performed, and location where they stayed or passed.

The data recorded were transferred to a personal computer and analyzed with the software EMCALC 2013 version 3.0B (Enertech Consultants Inc., Patterson, CA, USA). ELF-MF exposure was estimated as the arithmetic mean (AM) of all measurements throughout the period at work. All values are expressed in micro-tesla (µT).

### 2.4. Classification of ELF-MF Measurements

Based on a standardized checklist of information, the ELF-MF measurements (*n* = 942,884) logged every four seconds were categorized according to the following operational, job and activity factors and then compared.

- Type of semiconductor factory; fab and chip package factory
- Clean room vs non-clean room. The measurements recorded were dichotomized into clean and non-clean room.
- Type of job: operator, maintenance engineer, electric engineer and electric maintenance manager of. The electric maintenance manager performed administrative work in an office located outside a clean room.
- Type of operation: Operations within two types of semiconductor plant were categorized according to the operation to which workers were assigned.
- Microenvironments of location or job and activity performed: Locations where workers spent time or activities and jobs that workers performed were categorized: rest in clean room, rest outside cleanroom, near or passing electric devices assumed to be a source of ELF-MF and type of work activity, such as maintenance or operation.

### 2.5. Data Analysis

**Figure 2:**
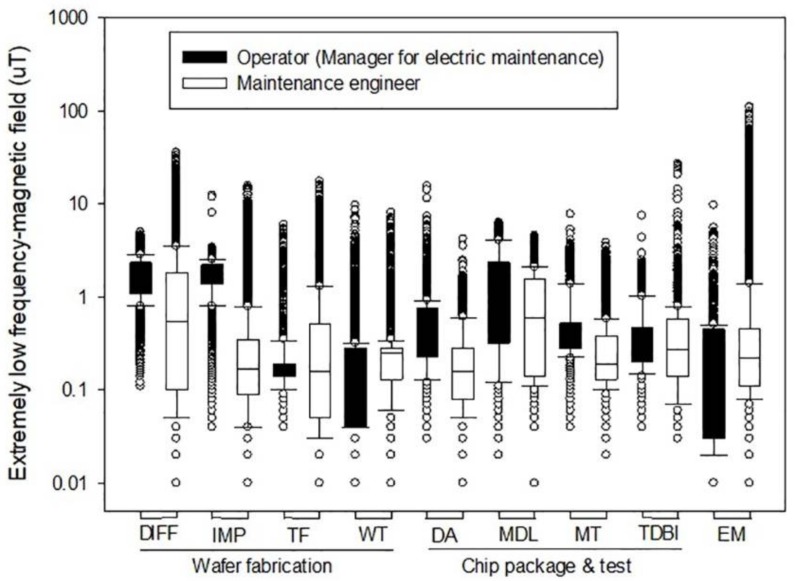
Distribution of extremely low frequency magnetic field (ELF-MF) exposure measurement by type of process and job (Abbreviations: DIFF: diffusion, IMP: ion implantation, TF: thin film, WT: wafer test, DA: die attach, MDL: module, MT: module test, TDBI: test during burn-in, EM: electric maintenance).

The ELF-MF exposure measurements were compared among the classifications of operational, job and activity characteristics described above. Although ELF-MF exposure levels for a given employee were measured every four seconds, resulting in a time-series type of data as in Figure 2, the AM of an employee in a different operation, job, and/or semiconductor plant was considered as an independent analysis unit. Here it is assumed that even in cases where an employee with the same monitoring device worked in different operations (or jobs), the exposure levels in one operation had nothing to do with those from other operations. With such an assumption, the differences in the AM levels of ELF-MF among types of semiconductor factory, types of operations and types of jobs were statistically tested through a Kruskal-Wallis rank test since a preliminary exploratory data analysis showed ELF-MF data recorded every four seconds were not normally distributed with an exceptionally large amount of outliers on the both the original and logarithmic scales and had different variabilities across operational or work factors (Figure 2). In contrast, ELF-MF levels of 117 semiconductor workers were found to be normally distributed (*p* < 0.0001).

ELF-MF peak exposure in this study is defined as intense ELF-MF exposures of a short duration greater than the full-shift AM of measurements monitored every four seconds within each worker (hereafter peak exposure), Since there has been no occupational exposure limit or criterion in the literature to define a high risk group due to excessive exposure to ELF-MF, relative ELF-MF exposure level for a semiconductor worker was qualitatively defined as low, moderate, and high level, based on quartiles of the overall ELF distribution regardless of factory, operation, job, and activity: low level for AM ELF-MF ≤ 50 percentile; moderate level for AM ELF-MF within a range of 50 to 75 percentile; high level for AM ELF-MF ≥ 75 percentile. Descriptive statistics and Kruskal-Wallis tests were performed using STATA 11 (STATA Corp., College Station, TX, USA). This study protocol was also approved by the Institutional Review Boards of Korea National Open University (ABN01-201502-11–02).

## 3. Results

A typical daily ELF-MF pattern for representative semiconductor workers is depicted in Figure 2, showing that ELF-MF exposure levels on a log scale were not normally distributed, with an asymmetric pattern in which an excessively large amount of high levels of ELF-MF exposure fell over 1.5 times the interquartile range above the third quartile (Q_3_). Several peak ELF-MF exposure patterns were observed among full-time semiconductor workers. For both operators and maintenance workers, peaks are generally relatively short exposure moments ranging from seconds to minutes, sometimes occurring with high frequency. Most semiconductor workers were found to be consistently exposed to peak levels.

ELF-MF exposure levels of semiconductor workers were indicated among types of operation and job within the type of semiconductor plant. Workers involved with diffusion (AM = 1.48 µT) and testing (AM = 1.46 µT) operations showed the highest average level of ELF-MF exposure, followed by module (AM = 1.13 µT) and electric maintenance (AM = 0.89 µT), which vary significantly according to the type of operation (*p* < 0.0001) (Table 1).

The average level of ELF-MF exposure of fab workers (AM = 0.56 µT) was found to be similar to that in the chip packaging factory (AM = 0.59 µT), which were significantly higher than the 0.28 µT of manager of electric maintenance workers (*p* < 0.0001) (Table 2).

Activities or locations where workers spent time were found to be the most substantial potential factor affecting ELF-MF exposure, even though other operations and job titles were found to contribute significantly to ELF-MF exposure as well. The ELF-MF exposure data inside a clean room (C/R) were found to be far higher than those monitored outside a clean room, including office environments (*p* < 0.0001) (Table 3).

**Figure 3:**
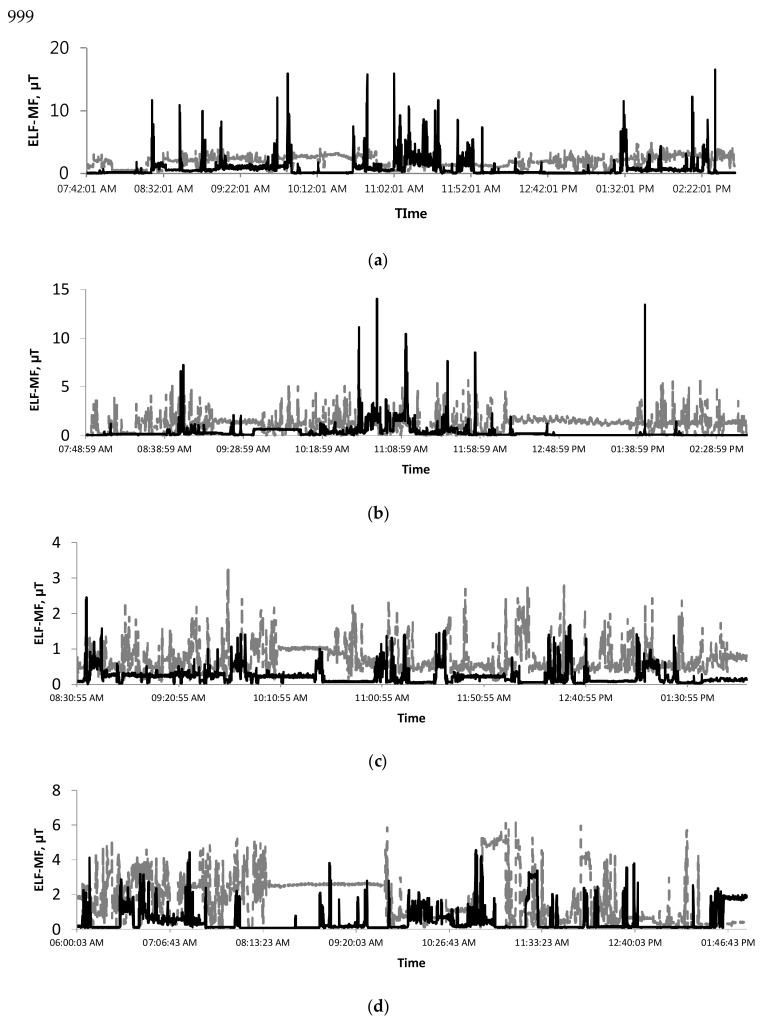
An example of ELF-MF exposure pattern of several semiconductor workers (solid black line: operator, OP; dotted grey line: maintenance engineer, ME). (**a**) Diffusion worker in fabrication (arithmetic mean, AM: OP = 1.93 µT, ME = 0.78 µT); (**b**) Thin-film worker in fabrication (AM: OP = 1.14 µT, ME = 0.34 µT); (**c**) Die-attach worker in package and test (AM: OP = 0.72 µT, ME = 0.22 µT); (**d**) Chip-mount worker in package and test (AM: OP = 1.75 µT, ME = 0.47 µT).

Our results indicated that ELF-MF exposure among workers performing tasks or passing for a short period near electric devices generating ELF-MF is high, compared to AM level (Figure 3).

**Figure 4:**
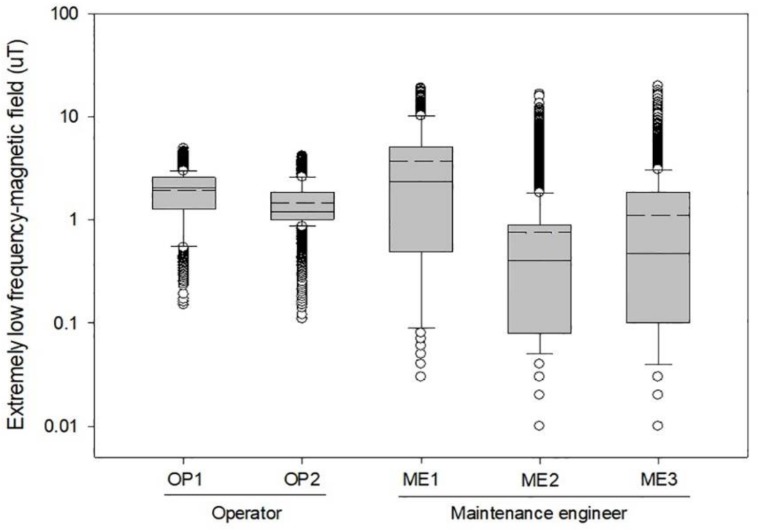
Distribution of full-shift extremely low frequency magnetic field (ELF-MF) exposure levels within each operator and maintenance engineer in the diffusion process (Dashed line: arithmetic mean).

We found that the same semiconductor worker can experience different levels of ELF-MF exposure on the same or different days depending on the tasks performed and the locations where they stayed or passed, if the same sources are present (Figure 4).

The qualitative ELF-MF exposure levels of semiconductor workers are relatively estimated based on the quartile distribution of ELF-MF. The estimated the highest quartile ELF-MF exposure groups includes operators in diffusion, ion implantation, module and testing operations, and maintenance engineers in diffusion, module and electric maintenance (Table 4).

## 4. Discussion

Using sophisticated magnetic field meters (EMDEX) to examine various patterns of time changes [14], we assessed the exposure to ELF-MF of semiconductor workers involved in fab and chip packaging operations that utilized a variety of electrical devices. The average levels of ELF-MF were substantially different among not only types of operations and jobs, but also activities or locations where workers spent time or passed near electric devices (Table 1, Table 2, Table 3 and Figure 2). Even among workers occupied in the same operation and job, their exposures vary substantially according to where they stay or spend time. It is clear that the ELF-MF levels experienced when working or passing for a short period near electric devices generating ELF-MF are typically higher than both AM and during other activities performed farther away from electric devices (Figure 3 and Figure 4). The ELF-MF exposure levels of fab workers in this study were found to be similar to those for fab workers reported to date. Two studies have assessed ELF-MF exposure among semiconductor workers in a clean room work environment in the semiconductor industry. Abdollahzadeh et al. reported on observations over the course of three years that indicated that device and area ELF-MF levels remained relatively constant during that period. The range of average ELF-MF exposure levels reported in 1991 (*n* = 50, 0.3–1.2 µT) [15] did not differ significantly from those in 1995 (*n* = 142, 0.2–1.0 µT) [8]. The ELF-MF exposure levels recently reported by Chung et al. were substantially similar to those reported in the 1990s. Diffusion showed the highest exposure level (AM = 1.14 µT), followed by photolithography (AM = 0.96 µT) [16]. In this study, the average ELF-MF exposure of chip packaging and test workers (AM = 0.59 µT, no. of workers = 45, no. of recordings = 359,862) were found to be similar to the levels of fab workers (AM = 0.56 µT). Our results are higher than the levels (front assembly AM = 0.22 µT, packaging AM = 0.10 µT) reported by Chung et al., who assessed the ELF-MF exposure levels of chip packaging workers even when sufficient numbers of processes and jobs were not covered [16]. Overall, the ELF-MF exposure levels of fab workers (no. of workers = 59, no. of observations = 475,015, mean = 0.56 µT, range = 0.01–35.36) were found to be generally higher than those reported in other jobs handling electric equipment or facilities such as electric engineer (AM = 0.275, *n* = 30), electric technician (AM = 0.366 µT, *n* = 27), electrician(AM = 0.366 µT, *n* = 150), fitter (AM = 0.298 µT, *n* = 25) and TV repairman (AM = 0.394 µT, *n* = 25) [13], even though lower than the average levels (New Zealand *n* = 5, 8.1 µT; LA *n* = 28, 1.75 µT; Korea *n* = 3, 3.46 µT) of exposure among welders [13,16].

Overall, the ELF-MF exposure levels of semiconductor workers can be assumed to have remained relatively constant, even if some operations showed different levels. The underlying parameter determining the stability of ELF-MF exposure is the stability of individual personal exposure, which in turn depends on the stability of ELF-MF levels from devices and areas. This estimation can be demonstrated by the results measured near process devices. Rosenthal and Abdollahzadeh reported ELF-MF area levels measured at aisles and versus distance from process devices. ELF-MF levels measured in the aisles of the workrooms ranged from 0.02 to 0.7 µT (*n* = 91) [15]. At two inches from the surfaces of various workroom devices, ELF-MF was 0.5 to 40 µT. ELF-MF showed the highest levels (mean = 27 µT, range = 10–40 µT) measured at two inches from sputter [15] and (10 µT and 15 µT) at two inches near a diffusion furnace. These results are very similar to those recently reported by Chung et al., who measured ELF-MF by distance for various devices (3 cm, 10 cm and 30 cm) [16]. The highest level of ELF-MF ranged from 280 to 860 µT monitored at three centimeters from diffusion equipment such as furnace. Our results indicate that great time trends have not been found in ELF-MF TWA exposure levels over time as reported in studies conducted to date in semiconductor operations [8].

Based on the review of ELF-MF levels reported in semiconductor factories, including our results, the stability of area and device levels and personal exposures may permit estimation of past ELF-MF exposures. Qualitative ELF-MF exposure scores (<0.2 µT, 0.2–0.5 µT, >0.5 µT) categorized through the model developed by Abdollahzadeh et al. were used to evalute the risk of spontaneous abortion among female fab workers [8]. This study’s results can be used to identify important factors related to various operations, jobs and microenvironment activities influencing ELF-MF exposure, and to classify semiconductor workers based on ELF-MF exposure level. Our results indicate that the location where workers spent time and the activity workers performed was the most important factor contributing to increases in ELF-MF exposure levels. When combining exposure per activity with time activity data from the diaries, activity near operation machines such as maintenance work was the most important contributor to ELF-MF exposure (Table 3 and Figure 2).

The ELF-MF exposure of semiconductor workers can be classified based on our results in order to evaluate chronic health effects in an epidemiologic study, including leukemia and brain cancer, which has been controversial in the past. Semiconductor workers who have performed tasks in diffusion, ion implantation, module and testing operations can be classified into the relatively high ELF-MF exposure group (Table 4). Any exposure group developed should have the same exposure distribution to which the individual subjects are exposed. This approach may always be challenging due to a lack of specificity of jobs, tasks or operations as they relate to the study subjects. It is common for semiconductor workers to be exposed to several peak levels for short periods of time as they perform tasks near electric devices.

Although the highest ELF-MF exposure level (109 µT) measured from electric engineers is below the reference levels at frequency of 60 Hz as recommended by the International Commission on Non-Ionizing Radiation Protection (ICNIRP) (ceiling limit = 417 µT) and by the ACGIH (ceiling limit = 1000 µT) [17,18], it is necessary to characterize peak exposure. European Union adopted the occupational exposure limit value and action level for ELF-MF based on the recommendations of ICNIRP in 2013 [19]. No studies have assessed peak ELF-MF exposure characteristics, such as number of peaks per hour, duration of a peak, maximum of level within a peak, average level within a peak etc in the semiconductor industry and in other industries. Peak exposures cannot be compared due to the lack of results reported. Further study is underway to characterize peak ELF-MF exposures of semiconductor workers based on operation, job and other occupational factors. In either epidemiologic study or exposure assessment study, measurements of ELF-MF are generally averaged over time without considering variation in the measurements monitored during a short period of time due to specific operation or job characteristics. Both TWA and peak exposure over a short period of time should be appropriately taken into account for evaluating possible health effects.

In 2002, IARC classified ELF-MF as possibly carcinogenic to humans. This classification was based on pooled analyses of epidemiological studies demonstrating a consistent pattern of a two-fold increase in childhood leukaemia associated with average exposure to residential power-frequency magnetic field above 0.3 to 0.4 µT [6]. In terms of adult cancers, many studies have shown controversial and inconclusive results [20,21]. Recently, a meta-analysis of 42 case-control studies suggests that ELF-EMFs are associated with cancer risk (OR = 1.08, 95% CI: 1.01–1.15), mainly in the United States (OR = 1.10; 95% CI: 1.01–1.20) and in residential exposed populations (OR = 1.18; 95% CI: 1.02–1.37) [22]. For exposure assessment of ELF-MF, many studies have estimated exposure intensity with interviews or distance between residential area and power line. Among 42 case-control studies, only 16 have conducted quantitative exposure assessment with ELF-MF dosimeter including EMDEX II and showed significant cancer risk with ELF-MF above 0.2 or 0.3 µT. In this study, the average levels of ELF-MF exposure of fab, chip packaging and electric maintenance workers were higher than 0.5 µT. In particular, workers of diffusion in fab and test in chip packaging showed the highest average exposure levels of 1.48 µT and 1.46 µT, respectively.

The associations observed in epidemiological studies between ELF-MF exposure and cancer are evaluated to be due to a promoter or progression effect rather than initiator, if the associations do in fact reflect a causal relation [5,23]. A Swedish study [5] also showed the possible interactive effect of occupational exposure to ELF-MF and to carcinogenic chemicals on the incidence of brain cancers in 2002. Occupational ELF-MF exposure using as reference those with an average mean below 0.13 µT (33rd percentile) was related to a weak increased risk of gliomas in the second exposure group (0.13–0.20 µT; RR = 1.12, 95% CI: 1.02–1.22) and in the third group (0.20–0.30 µT; RR = 1.12, 95% CI: 1.01–1.25). It has been well known that many chemicals including carcinogens are extensively used or generated in the wafer manufacturing process. It is common for fab workers to be exposed to chemicals and other carcinogens, such as shift work.

According to the first epidemiological study on the semiconductor industry in Korea, there was no significant increase of leukemia but, the incidence of non-Hodgkin’s lymphoma in females and thyroid cancer in males were significantly increased [24]. After that, the possibility of exposure to by-product volatile organic compounds including benzene in photolithography process was confirmed [25]. However, comprehensive ELF-MF exposure assessment in semiconductor industry has not been conducted in Korea. In 2018, a live-line working man who died of acute leukemia was recognized as the first occupational disease caused by ELF-MF in Korea. Although public concern on the ELF-MF exposure is increasing, there is no regulation on the ELF-MF to protect workers from potential cancer risk.

To protect semiconductor workers from high exposure of ELF-MF, the structural characteristics of semiconductor manufacturing plant should be considered. In general, fab contains a lot of electric equipment such as the steppers for photolithography, furnace and ion implant for diffusion, etching and cleaning devices which require electrical power. The specification of equipment investigated in this study showed that electrical voltage ranged from 120 V to 480 V and current ranged from 10 A to 1170 A with 60 Hz. The wires for providing electrical power to the electronic devices are installed like nets underneath the fab. So, operators and maintenance workers working in the fab are like working on electric blankets. In fact, we could confirm that the ELF-MF level is higher near the bottom of the fab. Within a fab, there may be areas with high levels of ELF-MF emissions near equipment with high current consumption. In order to reduce the exposure level of ELF-MF for operators, maintenance workers and electrical maintenance workers, the location of the residing table should be located far away from area with high levels of ELF-MF emissions. In addition, workers should be trained not to approach as close as possible to ELF-MF generating equipment.

It is unknown how representative our results may be, although ELF-MF exposures of workers from various semiconductor processes and jobs were assessed. The strength of our study is to report ELF-MF exposures levels by type of semiconductor operation and type of job, including maintenance work, and to identify significant factors influencing ELF-MF exposure. Kheifets et al. suggested that for future health studies regarding ELF-MF exposure, a more complete exposure assessment and investigation is needed [26]. Our results can be used to provide information on semiconductor operations and work with a relatively high probability of ELF-MF exposure and to identify semiconductor groups that are subject to higher ELF-MF exposure and classify ELF-MF exposure for epidemiologic study.

## 5. Conclusions

Semiconductor workers were generally found to be exposed to peak levels of ELF-MF far higher than the average level, with frequent occurrence for short period time when they performed tasks or stayed or passed near electric devices, even though there was no measurement exceeding established limits. Our results can be used to classify semiconductor workers based on ELF-MF exposure for epidemiologic study. Further study is needed to characterize high levels of peak ELF-MF to which semiconductor workers are frequently exposed for short periods of time.

## Figures and Tables

**Table 1:** ELF-MF exposure level by operation within the type of semiconductor plant.

Classification	Operation	No. of Workers	No. of Observations	AM, µT	SD, µT	GM, µT	GSD	Range, µT
Fabrication *	CMP	6	44,242	0.14	0.29	0.09	2.47	0.01–16.83
	Photolithography	6	53,883	0.53	0.46	0.37	2.54	0.02–21.97
	Etch	11	98,151	0.53	0.64	0.37	2.40	0.01–23.52
	Diffusion	8	58,672	1.48	2.00	0.64	4.50	0.01–35.36
	Ionimplantation	8	60,650	0.76	0.95	0.32	4.18	0.01–15.67
	Thin-film	8	66,598	0.43	0.77	0.18	3.64	0.01–17.31
	Wafertest	11	92,819	0.21	0.29	0.14	2.62	0.01–9.63
	Total	58	475,015	0.56	0.99	0.25	3.61	0.01–35.36
Chip packaging *	Dieattach	8	69,152	0.46	0.38	0.33	2.41	0.01–15.39
	Module	5	47,401	1.13	1.16	0.59	3.56	0.01–6.27
	Moduletest	11	105,017	0.39	0.43	0.27	2.24	0.02–7.71
	TDBI	14	105,812	0.39	0.39	0.28	2.32	0.03–26.72
	Test	7	32,480	1.46	0.90	1.24	1.80	0.08–21.92
	Total	45	359,862	0.59	0.71	0.36	2.73	0.01–26.72
Electric maintenance *	Maintenance	12	91,481	0.89	3.11	0.27	3.55	0.01–109.00
	Management	2	16,526	0.28	0.42	0.13	4.06	0.01–9.66
	Total	14	108,007	0.80	2.88	0.24	3.73	0.01–109.00

Abbreviations: CMP: chemical mechanical polishing, TDBI: test during burn-in, AM: arithmetic mean, SD: standard deviation, GM: geometric mean, GSD: geometric standard deviation. * *p* < 0.0001, Kruskal-Wallis rank test by operations.

**Table 2:** ELF-MF exposure level by type of job within the type of semiconductor plant.

Classification	Type of Job	No. of Workers	No. of Observations	AM, µT	SD, µT	GM, µT	GSD	Range, µT
Fabrication *	Operator	13	106,987	0.68	0.85	0.27	4.23	0.01–12.31
	M/E	45	368,028	0.53	1.02	0.25	3.43	0.01–35.36
Chip packaging *	Operator	22	155,994	0.82	0.86	0.54	2.52	0.02–21.92
	M/E	23	203,868	0.42	0.51	0.26	2.57	0.01–26.72
Electric maintenance *	Engineer	12	91,481	0.89	3.11	0.27	3.55	0.01–109.0
	Manager	2	16,526	0.28	0.42	0.13	4.06	0.01–9.66

Abbreviations: M/E: maintenance engineer, AM: arithmetic mean, SD: standard deviation, GM: geometric mean, GSD: geometric standard deviation. * *p* < 0.0001, Kruskal-Wallis rank test by jobs.

**Table 3:** ELF-MF exposure level by type of activity within the type of semiconductor plant.

Classification	Type of Activity or Location	No. of Observations	AM, µT	SD, µT	GM, µT	GSD	Range, µT
Fabrication *	Resting outside clean room	51,953	0.27	0.25	0.17	2.73	0.01–2.51
	Resting inside clean room	19,632	0.77	0.54	0.60	2.11	0.03–2.65
	Operation work	91,603	0.66	0.85	0.26	4.28	0.01–3.45
	Maintenance work	14,952	3.34	3.24	2.32	2.36	0.05–35.36
	Near or passing process machine	296,875	0.43	0.60	0.22	3.27	0.01–10.70
Chip packaging *	Resting outside clean room	34,056	0.23	0.47	0.13	2.20	0.03–2.87
	Resting inside clean room	11,353	0.55	0.60	0.37	2.22	0.09–2.58
	Operation work	130,620	0.73	0.60	0.54	2.21	0.02–4.41
	Maintenance work	16,294	2.19	1.59	1.77	1.93	0.03–26.72
	Near or passing process machine	167,539	0.41	0.42	0.27	2.45	0.01–4.55
Electric engineer *	Resting outside clean room	2735	0.34	0.27	0.26	2.29	0.02–3.48
	Resting inside clean room	994	1.17	0.79	0.69	3.62	0.01–2.87
	Maintenance work	3421	11.43	11.06	7.77	2.49	0.54–109.0
	Near or passing process machine	84,331	0.48	0.92	0.24	2.96	0.01–9.99
Manager of electric maintenance *	Resting outside clean room	1072	0.15	0.15	0.07	3.81	0.02–0.35
	Office	15,207	0.25	0.23	0.13	3.89	0.01–1.95
	Near or passing electric device	247	2.65	1.62	2.15	1.97	0.60–9.66

Abbreviations: AM: arithmetic mean, SD: standard deviation, GM: geometric mean, GSD: geometric standard deviation. * *p* < 0.0001, Kruskal-Wallis rank test by activities.

**Table 4:** Classification of semiconductor workers based on the distribution of ELF-MF exposure level.

Classification	Operation	Type of Job	No of Samples	AM, uT	SD, uT	GM, uT	GSD	Range, uT	Relative Exposure Group ^1^
Fabrication	CMP	M/E	44,242	0.14	0.29	0.09	2.47	0.01–16.83	Low
	Photolithography	M/E	53,883	0.53	0.46	0.37	2.54	0.02–21.97	Moderate
	Etch	M/E	98,151	0.53	0.64	0.37	2.40	0.01–23.52	Moderate
	Diffusion	Operator	16,869	1.69	0.81	1.48	1.71	0.11–4.93	High
		M/E	41,803	1.40	2.30	0.46	5.10	0.01–35.36	High
	Ion implantation	Operator	17,099	1.74	0.68	1.50	1.97	0.04–12.31	High
		M/E	43,551	0.37	0.74	0.17	3.20	0.01–15.67	Low
	Thin film	Operator	8395	0.24	0.40	0.18	1.86	0.04–5.93	Low
		M/E	58,203	0.45	0.81	0.18	3.90	0.01–17.31	Moderate
	Wafer test	Operator	64,624	0.19	0.25	0.12	2.66	0.01–9.63	Low
		M/E	28,195	0.27	0.36	0.19	2.34	0.01–7.99	Low
Chip packaging	Die attach	Operator	51,536	0.53	0.39	0.41	2.10	0.03–15.39	Moderate
		M/E	17,616	0.25	0.26	0.16	2.41	0.01–4.17	Low
	Module	Operator	18,601	1.46	1.48	0.76	3.67	0.02–6.27	High
		M/E	28,800	0.91	0.82	0.50	3.38	0.01–4.55	High
	Module test	Operator	28,799	0.56	0.52	0.43	1.99	0.04–7.71	Moderate
		M/E	76,218	0.32	0.37	0.23	2.17	0.02–3.81	Moderate
	TDBI	Operator	24,578	0.43	0.34	0.33	2.00	0.04–7.43	Moderate
		M/E	81,234	0.37	0.40	0.26	2.40	0.03–26.72	Moderate
	Test	Operator	32,480	1.46	0.90	1.24	1.80	0.08–21.92	High
Electric maintenance	Maintenance	M/E	91,481	0.89	3.11	0.27	3.55	0.01–109.0	High
	Management	Manager	16,526	0.28	0.42	0.13	4.06	0.01–9.66	Low

Abbreviations: CMP: chemical mechanical polishing, TDBI: test during burn-in, M/E: maintenance engineer, AM: arithmetic mean, SD: standard deviation, GM: geometric mean, GSD: geometric standard deviation. ^1^ Low: AM < 0.3 µT, Moderate: 0.3 ≤ AM < 0.65 µT, High: AM ≥ 0.65 µT.
